# ENVI-met validation data accompanied with simulation data of the impact of facade greening on the urban microclimate

**DOI:** 10.1016/j.dib.2022.108200

**Published:** 2022-04-22

**Authors:** Hayder Alsaad, Maria Hartmann, Rebecca Hilbel, Conrad Voelker

**Affiliations:** Bauhaus-University Weimar, Department of Building Physics, Weimar, Germany

**Keywords:** Measurements, Simulations, ENVI-met, Living wall, green facade

## Abstract

This dataset consists mainly of two subsets. The first subset includes measurements and simulation data conducted to validate the simulation tool ENVI-met. The measurements were conducted at the campus of the Bauhaus-University Weimar in Weimar, Germany and consisted of recording exterior air temperature, globe temperature, relative humidity, and wind velocity at 1.5 m at four points on four different days. After the measurements, the geometry of the campus was modelled and meshed; the simulations were conducted using the weather data of the measurements days with the aim of investigating the accuracy of the model.

The second data subset consists of ENVI-met simulation data of the potential of facade greening in improving the outdoor environment and the indoor air temperature during heatwaves in Central European cities. The data consist of the boundary conditions and the simulation output of two simulation models: with and without facade greening. The geometry of the models corresponded to a residential buildings district in Stuttgart, Germany. The simulation output consisted of exterior air temperature, mean radiant temperature, relative humidity, and wind velocity at 12 different probe points in the model in addition to the indoor air temperature of an exemplary building. The dataset presents both vertical profiles of the probed parameters as well as the time series output of the five-day simulation duration. Both data subsets correspond to the investigations presented in the co-submitted article [1].

## Specifications Table

**Table:** 

Subject	Civil and Structural Engineering
Specific subject area	Measurements and simulations to validate ENVI-met Simulations of the potential of facade greening in mitigating the effects of heatwaves
Type of data	Tables Figures Comma-separated values (CSV) files ENVI-met geometry files (INX files) ENVI-met material files (edb files)
How the data were acquired	Empirical measurements Numerical simulations using ENVI-met (version 4.4)
Data format	Raw, formatted
Description of data collection	Empirical measurements were conducted at four points at the campus of the Bauhaus-University Weimar in Weimar, Germany using a mobile measurement station. The station consisted of sensors to measure air temperature, globe temperature, relative humidity, and air velocity. The numerical simulations were conducted using ENVI-met. Two sites were simulated: the campus of the Bauhaus-University Weimar with the goal of validating the model, and a residential buildings district in Stuttgart, Germany with the goal of quantifying the impact of facade greening on the microclimate during heatwaves.
Data source location	Institution: Bauhaus-University Weimar City/Town/Region: Weimar Country: Germany Latitude and longitude: 50°58′55"N 11°19′20"E
Data accessibility	The dataset is available on Mendeley Data DOI: 10.17632/9bm76zcw28.1 URL: https://data.mendeley.com/datasets/9bm76zcw28/1
Related research article	H. Alsaad, M. Hartmann, R. Hilbel, C. Voelker, The potential of facade greening in mitigating the effects of heatwaves in Central European cities, Building and Environment 216 (2022) 109021. 10.1016/j.buildenv.2022.109021.

## Value of the Data

• This dataset provides empirical measurements values that can be used to validate other CFD simulation models.
• The presented ENVI-met validation data allow other researchers to conduct further simulations to explore the potential of the different models and settings embedded in ENVI-met.
• The presented simulation dataset allows further analysis and assessment of the impact of facade greening on different environmental parameters during heatwaves.

## 1 Data Description

The data consists of two subsets: the first subset presents the validation data of ENVI-met and the second subset consists of the simulations of the impact of facade greening on the urban microclimate during heatwaves.

### 1.1 Data subset 1: ENVI-met validation

This subset includes both empirical measurements and simulation data generated using ENVI-met. The raw data of this subset are provided with this article as comma-separated values (CSV) files. Each file name starts with ‘DataSubset1’ to distinguish them from the data files that belong to the second subset. Afterwards, the presented parameter is mentioned in the file name, namely either ‘Simulations’ or ‘Measurements’. The third part of the file name corresponds to the location where the measurements or the simulation data were probed. This ranges from P1 to P4, corresponding to the four investigated points. More information about these four points is presented in the following section (Experimental design, materials and methods). The fourth and final part of the file name corresponds to the presented variable in the CSV file. For example, a file labelled DataSubset1_Measurements_P1_BoundaryConditions corresponds to the measured boundary conditions (weather data) during the measurements conducted in P1. Similarly, the file name DataSubset1_Simulations_P2_Output indicates the exported simulated values at P2. Further details about the presented parameters and their units are introduced within the CSV file. In addition to the CSV files, the attached data include the geometry file of the simulation model (INX file) and the ENVI-met material file (edb file) used for conducting the simulations.

### 1.2 Data subset 2: The impact of facade greening

**Fig. 1:**
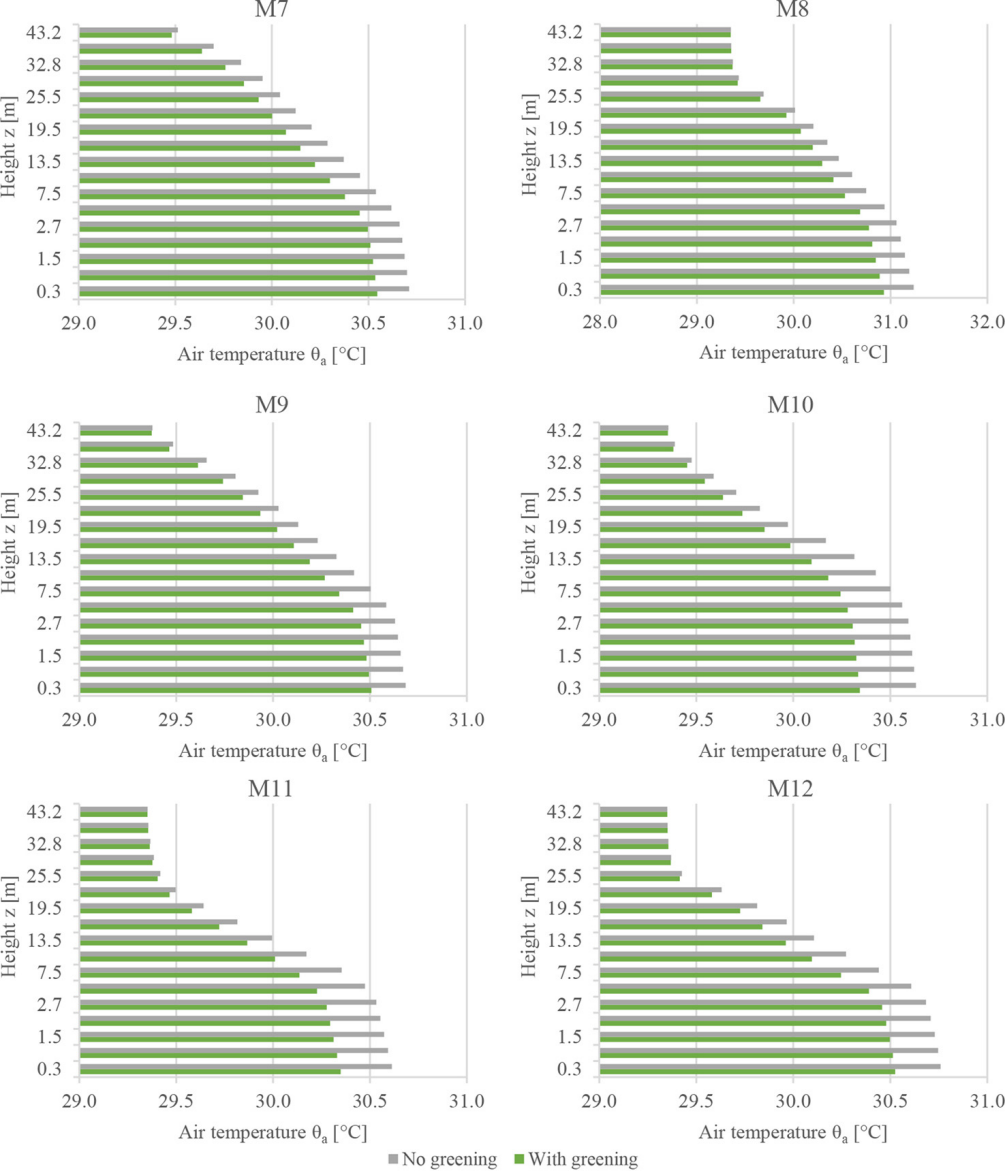
Vertical profiles of air temperature at the points M7 to M12 on the last day of the simulations at 5:00 pm

**Fig. 2:**
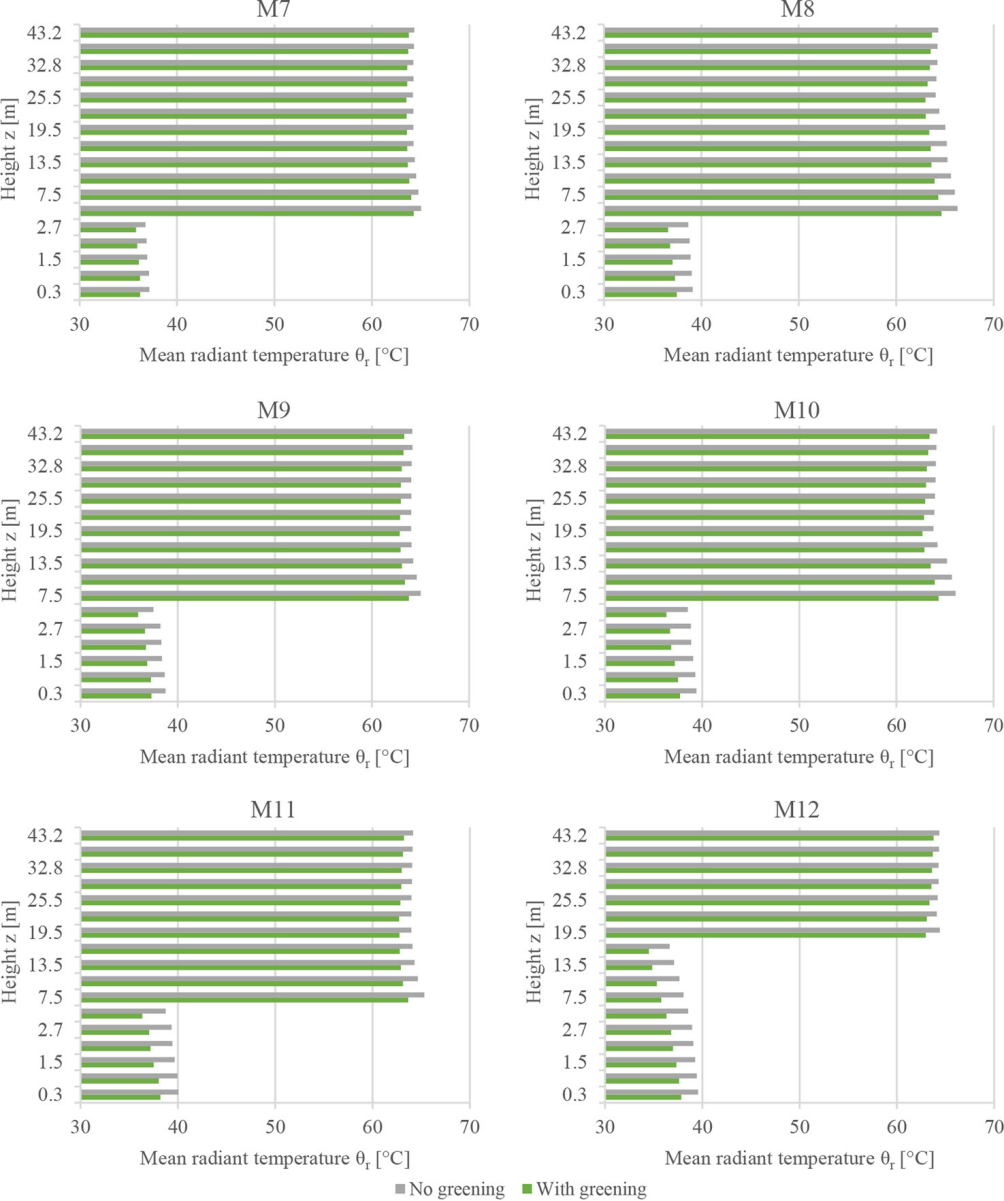
Vertical profiles of the mean radiant temperature at the points M7 to M12 on the last day of the simulations at 5:00 pm

**Fig. 3:**
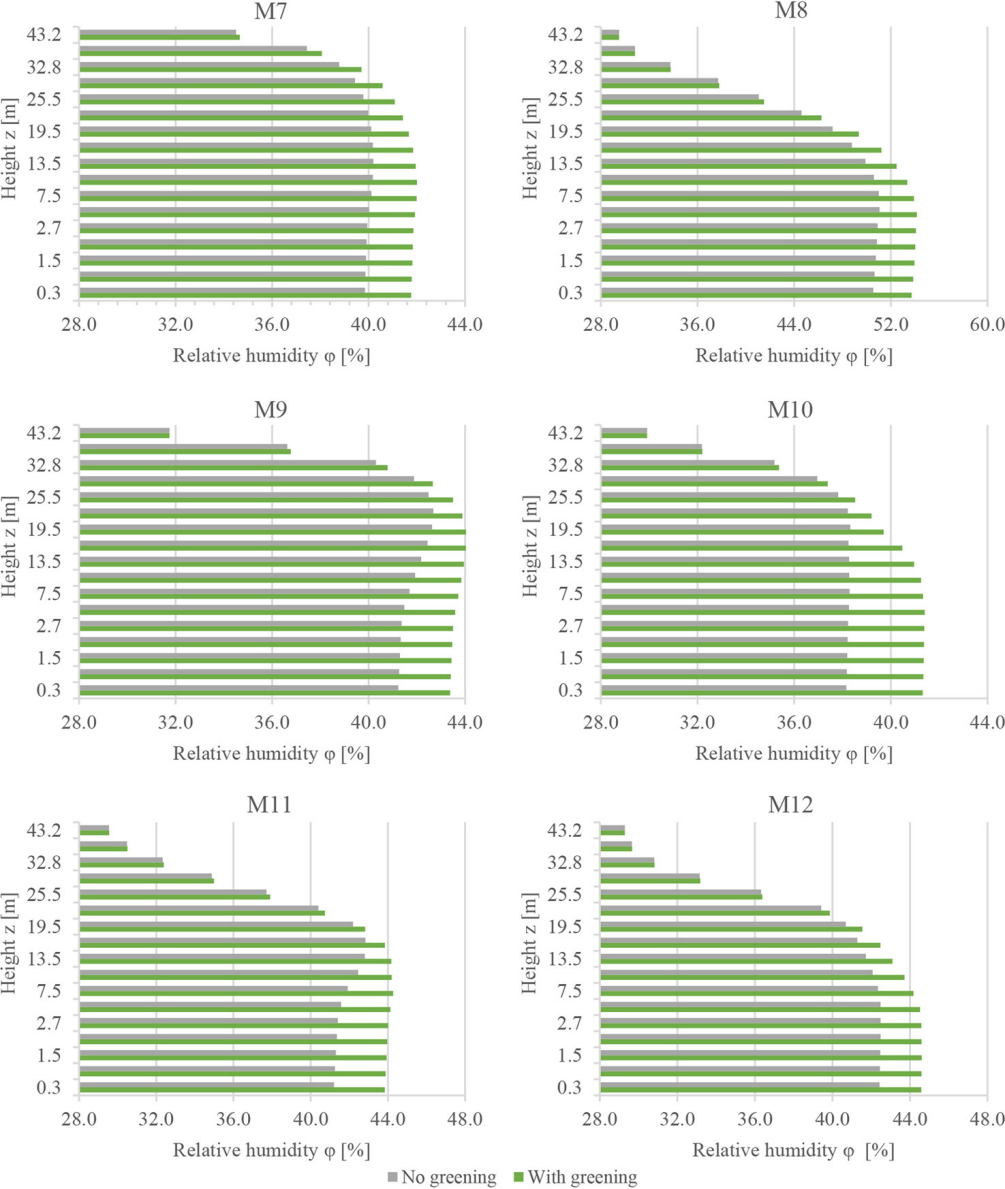
Vertical profiles of the relative humidity at the points M7 to M12 on the last day of the simulations at 5:00 pm

**Fig. 4:**
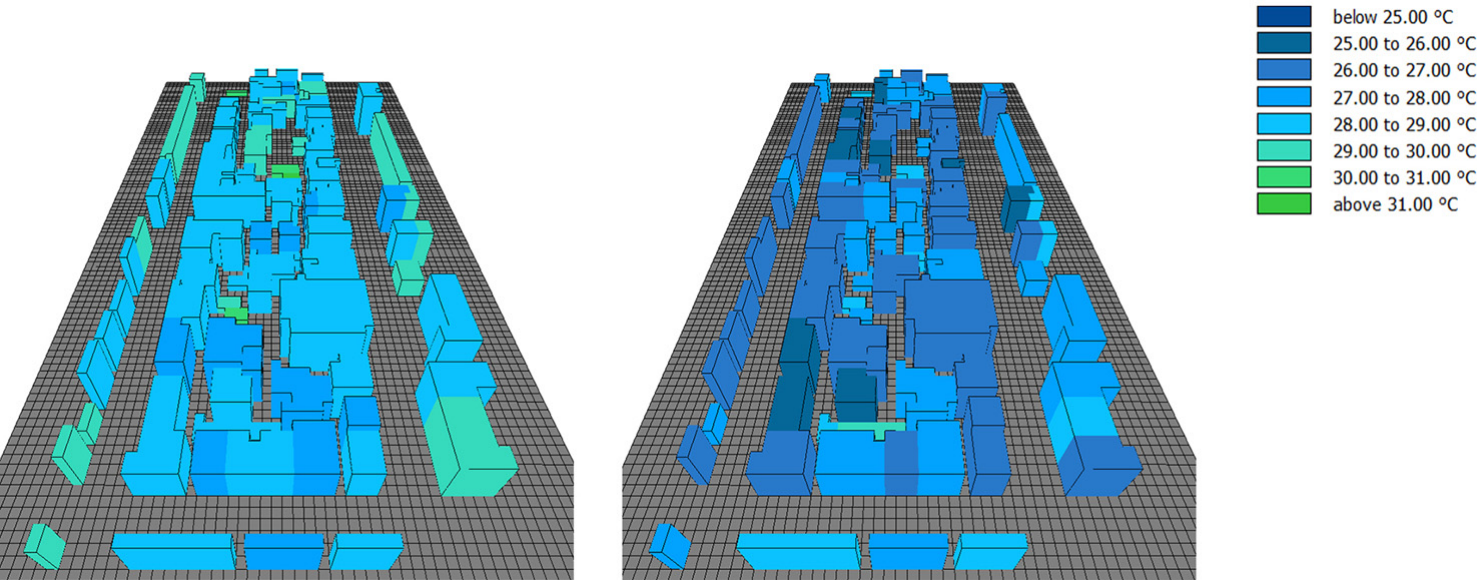
Interior air temperature without (left) and with facade greening (right) on the last day of the simulations at 5:00 pm

**Fig. 5:**
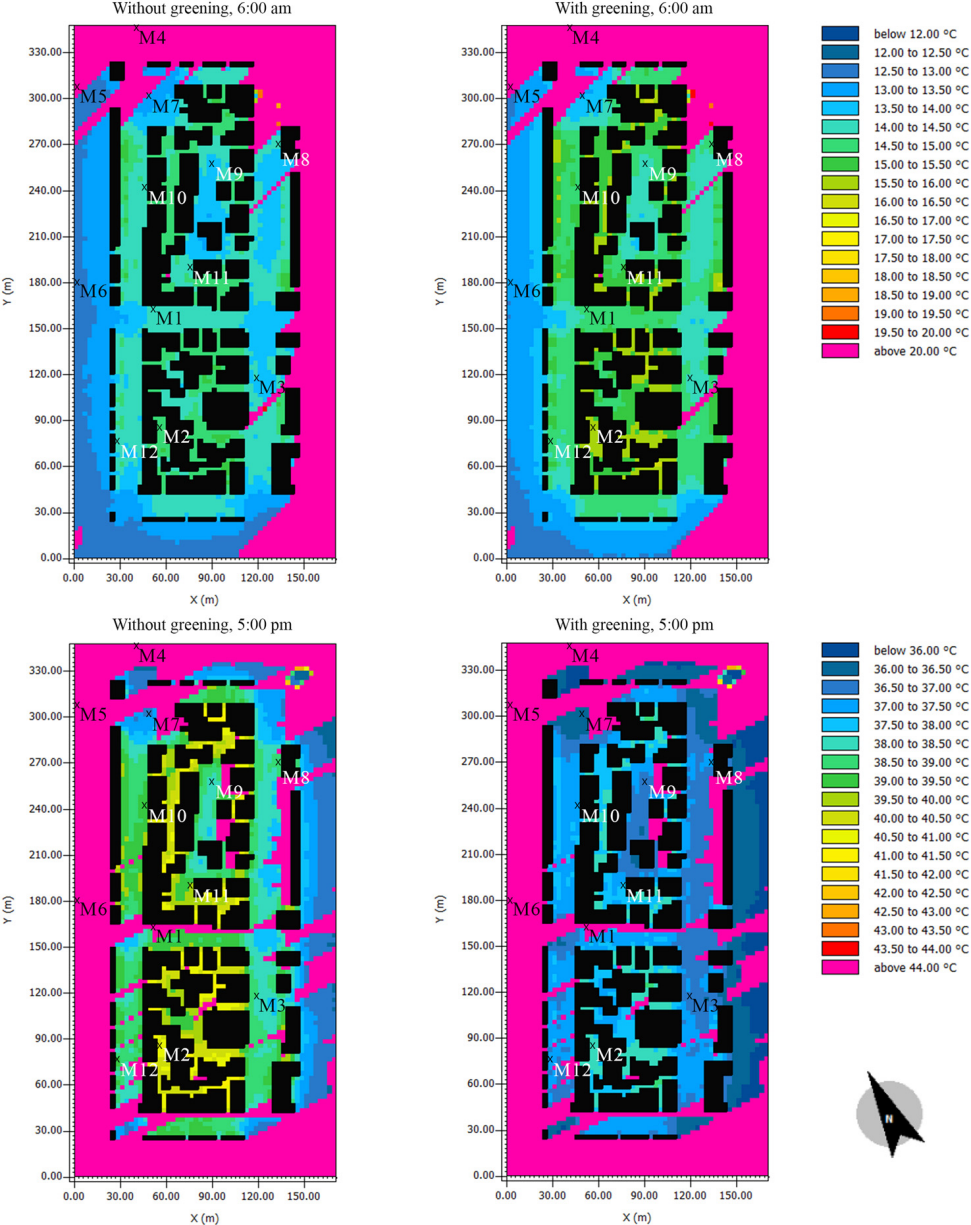
Mean radiant temperature without (left) and with facade greening (right) on the last day of the simulations at 6:00 am and 5:00 pm (horizontal section at the height of 1.5 m)

This subset presents the full dataset of the conducted numerical simulations aiming to assess the potential of facade greening in reducing the effects of heatwaves in Central European cities. While the supported article [1] presented analysis, discussions, and insights into the data, it was not possible to present all the data there due to length limitations. Therefore, the supported article presented the results from only six probe locations in the simulation domain. In the present article, however, the simulated values are presented for an additional six locations. Fig. 1, Fig. 2, Fig. 3 present the vertical profiles of the simulated air temperature, mean radiant temperature, and relative humidity, respectively, at the additional six locations (M7 to M12) for both simulation models: without and with facade greening. The height values illustrated on the y-axis correspond to the centre of each cuboid cell in the simulation model. Fig. 4 illustrates the difference in the indoor air temperature when facade greening is implemented. These Figs 1–4 present the exported data during the last simulation day (24.06.2019) at 5:00 pm. Fig. 5 indicates the deviating effect of facade greening on the mean radiant temperature between early morning and the afternoon.

The simulation data from all 12 probe points are provided as CSV files. These files include vertical profiles of air temperature, mean radiant temperature, relative humidity and air velocity at the last simulation day at 5:00 pm together with the time series output of the five-day simulation duration probed at 1.5 m. An additional CSV file is provided for the time series output of the indoor air temperature values of the exemplary building presented in the supported article [1]. Moreover, the provided files include the input boundary conditions, the geometry files of the simulation models (INX files) and the ENVI-met material file (edb file). The files names follow the same logic used for naming the files of data subset 1. For example, a file named DataSubset2_Simulations_M1_Profiles corresponds to the vertical profiles of all the environmental parameters probed at the location M1.

## 2 Experimental Design, Materials and Methods

**Fig. 6:**
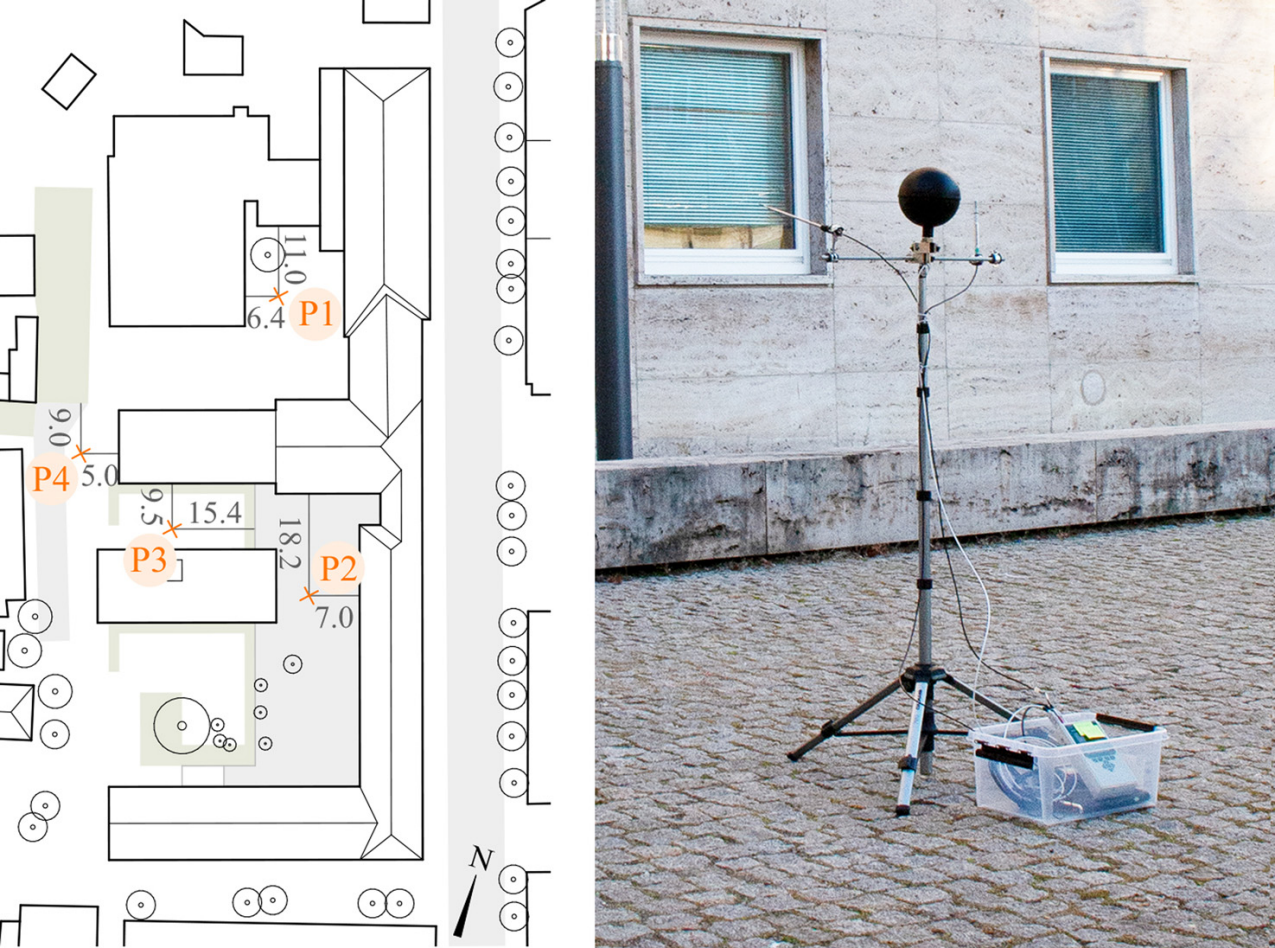
The measurements site and the measurements points (left) and the mobile measurement station at P2 (right)

Both presented data subsets include numerical simulations data acquired from the computational fluid dynamics (CFD) tool ENVI-met [2,3]. The implementation of ENVI-met in numerical assessments to evaluate the urban microclimate has been growing lately. Moreover, by coupling ENVI-met with other simulation tools, further aspects such as building energy performance [4] and the hygrothermal performance of building assemblies [5], [6], [7] can be investigated. Yet, before the simulations, the tool and its selected settings must first be validated against measured data. The measurements and simulations of the validation work comprise the Data subset 1 mentioned in the previous section. The measurements were conducted at the campus of the Bauhaus-University Weimar in the fall of 2020. The data were collected at four points (P1 to P4; Fig. 6, left) on four separate days (P1: 18.11.2020, P2: 24.11.2020, P3: 27.11.2020, and P4: 1.12.2020). The measurements were conducted at points P1, P2, and P3 from 3:00 pm to 6:00 pm. At P4, however, data collection was ended at 5:24 pm due to rain. The measurements were conducted using a mobile measurement station with a height of 1.5 m (Fig. 6, right). The station consisted of NTC type N air temperature sensor with an accuracy of ±0.2 K and a resolution of 0.01 K, a globe thermometer with a 150 mm black globe with a Pt100 element with an accuracy of ±(0.3 + 0.005|T|) K and a resolution of 0.01 K, a capacitive relative humidity sensor with an accuracy of ±2% RH and a resolution of 0.1%, and an omnidirectional thermos-anemometer with an accuracy of ±(3% of the measured value +1% of final value + 0.5% of the measured value/K) and a resolution of 0.001 m/s. All the sensors were connected to an Almemo portable data logger with a sampling interval of two minutes; the sensors and the data logger are from the German manufacturer Ahlborn.

**Fig. 7:**
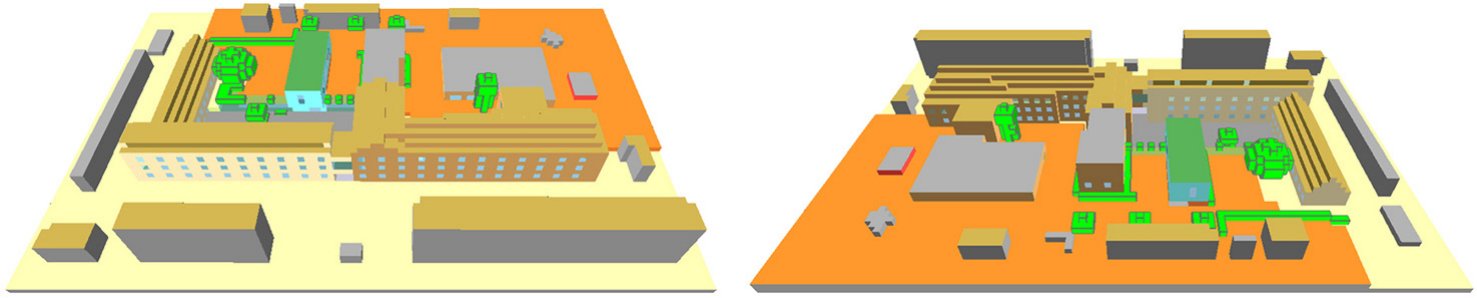
The geometry of the validation model viewed from the north-east (left) and the south-west (right)

**Fig. 8:**
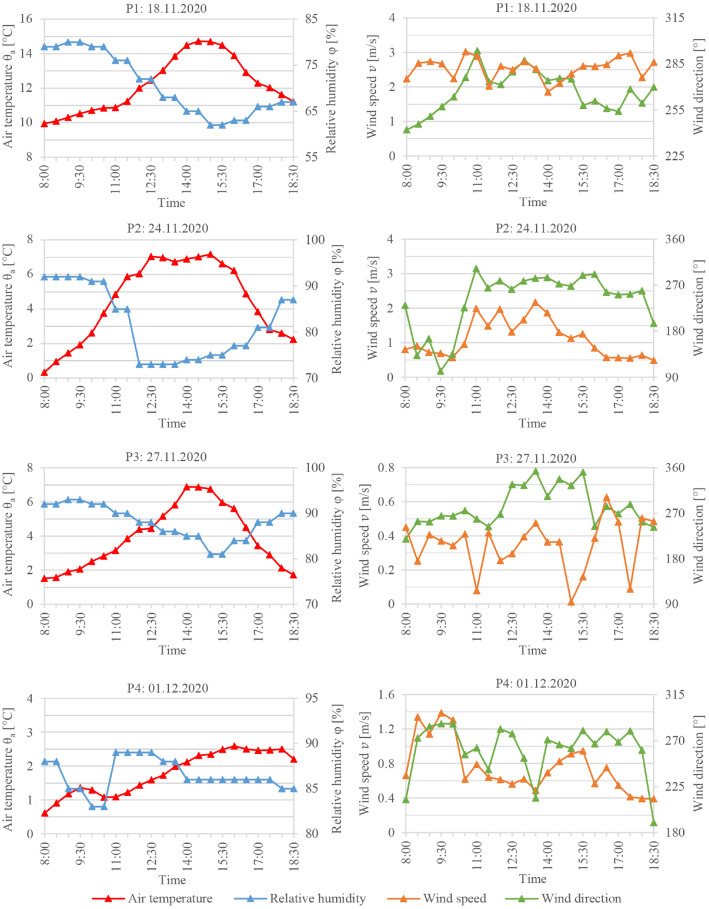
The boundary conditions at the inflow for the four validation days acquired from the measured weather parameters

After conducting the measurements, the site was modelled in ENVI-met to run the validation simulations. The modelling started with converting two-dimensional maps from OpenStreetMap into a three-dimensional model using the ENVI-met modelling tool Monde. Afterwards, details such as roof shapes and windows were added using another ENVI-met modelling tool, namely Spaces. Since the ENVI-met implements an orthogonal Arakawa C-grid, sloped roofs had to be approximated as stepped roofs (Fig. 7). The Database Manager was then used to define the material properties which were assigned to the surfaces in the domain in Spaces. Both building details and materials were adopted from the university archives and can be found in the attached INX and edb files. Further details such as the location and size of trees were based on personal observations and site measurements. As boundary conditions, the Forcing Manager was implemented to create the so-called full-forcing files from the measured weather parameters, which included air temperature, relative humidity, wind speed, and wind direction (Fig. 8). Radiation values were taken from the default location-specific values provided by ENVI-met. Table 1 presents the settings implemented in the validation model.

**Table 1:** The details and settings of the validation model

Software and version	ENVI-Met 4.4
Computation domain	124 × 192 × 121 m
Basic cell size	2 × 2 × 2 m (dx, dy, dz)
Mesh growth rate	20% on the vertical direction starting from 25 m
Number of grid cells	62 × 96 × 25 (W x L x H)
Near-ground grid cells	Divided into five sub-cells
Topography	Included; lowered model ground to -4 m at some parts of the domain
Simulation site	Weimar, Germany, 50°58′55"N 11°19′20"E
Weather data (air temperature, wind speed, and wind direction)	Weather station of the Department of Building Physics, Bauhaus-University Weimar
Weather data (relative humidity)	Weather station of the Thuringia Department of Environment, Mining, and Nature Protection
Weather data (longwave radiation, direct shortwave radiation, and diffuse shortwave radiation)	Default location-specific values provided by ENVI-met
Lateral boundaries setup	Full forcing; 30 min time steps at the inflow
Indoor conditions	Constant, 20 °C
Simulation time step	2 s at the initialization 1 s throughout the rest of the simulation time
Simulation time span	8 am to 6:30 pm on 18.11.2020, 24.11.2020, 27.11.2020, and 01.12.2020
Data output	15 min interval
Turbulence model	E-epsilon 1.5 order turbulence closure

**Fig. 9:**
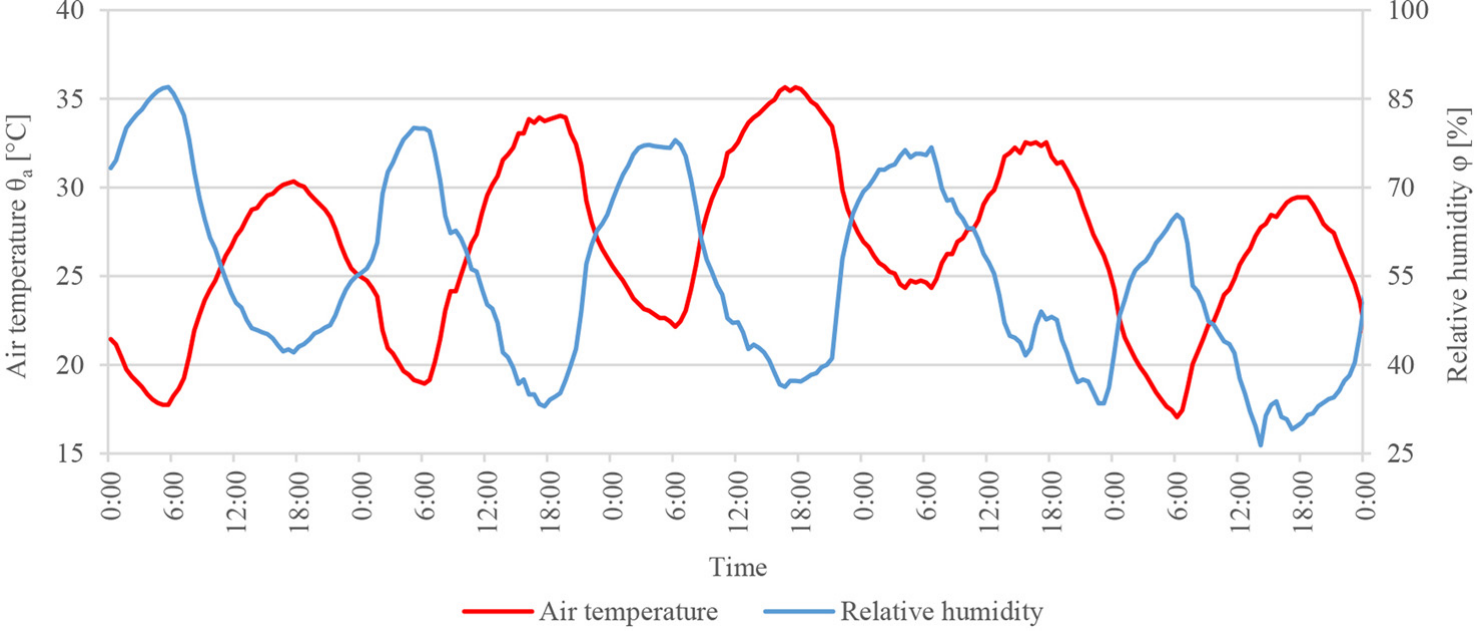
The air temperature and relative humidity in the boundary conditions file for the facade greening assessment models

**Fig. 10:**
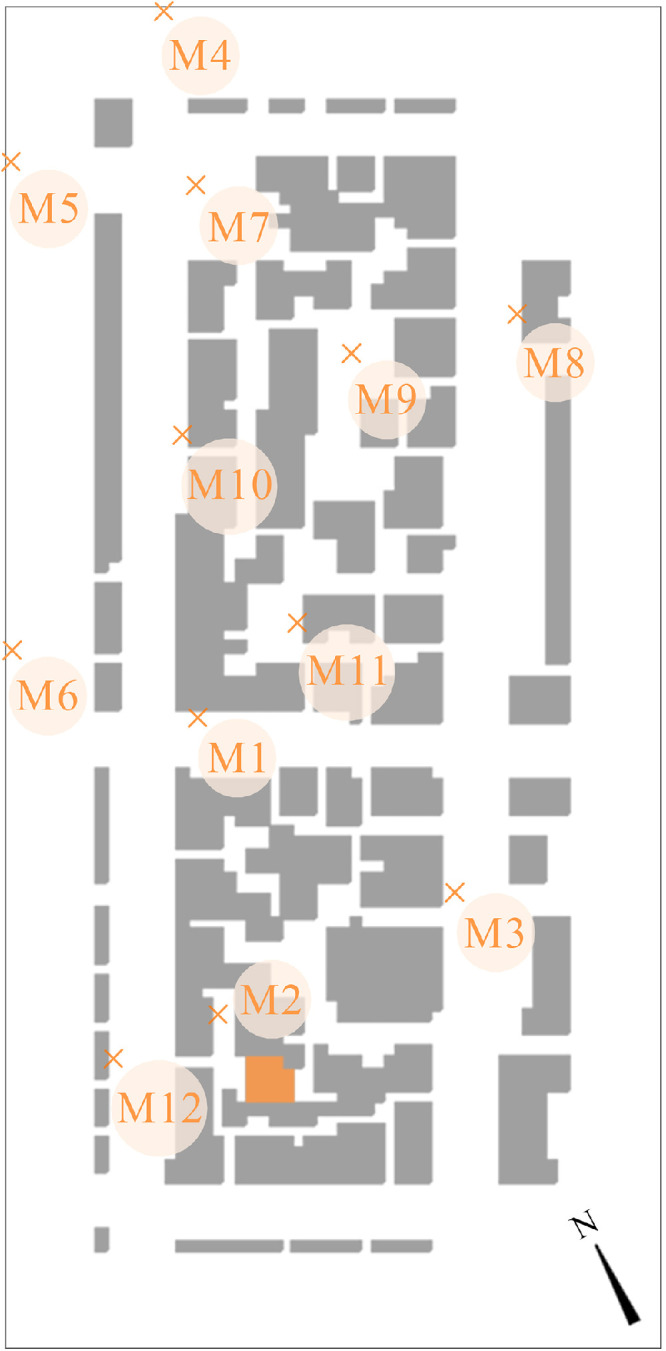
The position of the probe points within the model. The marked building indicates the location where the indoor air temperature was probed

After model validation, numerical simulations were conducted to investigate the impact of facade greening on the urban microclimate by comparing a model with facade greening to a reference model with no greening. The input and output of these simulations comprise the Data subset 2 mentioned in the previous section. The simulated area corresponded to a residential block in Stuttgart, Germany. Table 2 presents the details and settings of the simulated models. The modelling process followed the same approach used for the validation model. However, due to the lack of information, the domain had to be simulated using generic material as can be seen in the attached INX and edb files. Moreover, the building heights were not available and were estimated based on a story height of 3 m. To simplify the geometry, the form of the pitched roofs was neglected; half the height of the pitched roof was added to the building's height instead. To simulate the facade greening case, the properties of the living wall system developed by Londong and Aicher [8] were implemented. The detailed attributes of this greening system can be found in the attached Database Manager file. As boundary conditions, a full-forcing file was generated and included variable values of air temperature and relative humidity taken from the weather data provided by the weather station S-Mitte Schwabenzentrum (Fig. 9). Wind speed and wind direction, on the other hand, were set to a constant of 1.80 m/s and 156.4° clockwise from the north, respectively. These values were set as constant to ensure the stability of the simulations due to the relatively large domain. After all the settings were defined, the simulations were conducted in the solver Envi-Core and the output was accessed using the post-processer Leonardo. To quantify the impact of facade greening, air temperature, mean radiant temperature, relative humidity and wind speed were probed at 12 locations shown in Fig. 10. Moreover, the impact of greening on the indoor air temperature was probed at an exemplary building adjacent to the courtyard of M2 (marked in orange in Fig. 10).

**Table 2:** The details and settings of facade greening simulation models

Software and version	ENVI-Met 4.4
Computation domain	171 × 348 × 62.75 m
Basic cell size	3 × 3 × 3 m (dx, dy, dz)
Mesh growth rate	20% on the vertical direction starting from 27 m
Number of grid cells	57 × 116 × 19 (W x L x H)
Near-ground grid cells	Divided into five sub-cells
Topography	Not included
Simulation site	Stuttgart, Germany, 48°45′56"N 9°10′12"E
Number of simulated models	Two: with and without facade greening
Facade greening	On three sides; only the northeast facade was left bare
Facade greening construction	300 mm plants, 225 mm peat and biochar substrate mixture, 12 mm polyethylene (PE) board, and 50 mm air gap
Weather data (air temperature, wind speed, wind direction, and relative humidity)	Weather station S-Mitte Schwabenzentrum of the Stuttgart Office for Environmental Protection
Weather data (longwave radiation, direct shortwave radiation, and diffuse shortwave radiation)	Default location-specific values provided by ENVI-met
Lateral boundaries setup	Full forcing; 30 min time steps at the inflow
Indoor conditions	Variable (depending on outdoor conditions)
Simulation time step	2 s at the initialization 1 s throughout the rest of the simulation time
Simulation time span	24 to 28 June 2019
Data output	30 min interval
Turbulence model	E-epsilon 1.5 order turbulence closure

## Ethics Statements

No ethical issues are associated with this work.

## Data Availability

The dataset is available on Mendeley Data [9].

## CRediT authorship contribution statement

**Hayder Alsaad:** Conceptualization, Methodology, Formal analysis, Investigation, Data curation, Writing – original draft, Writing – review & editing, Supervision, Project administration. **Maria Hartmann:** Methodology, Software, Formal analysis, Investigation, Data curation, Writing – original draft, Writing – review & editing, Visualization. **Rebecca Hilbel:** Methodology, Software, Validation, Formal analysis, Investigation, Data curation, Writing – review & editing, Visualization. **Conrad Voelker:** Methodology, Resources, Writing – review & editing, Supervision, Project administration, Funding acquisition.

## Declaration of Competing Interest

The authors declare that they have no known competing financial interests or personal relationships that could have appeared to influence the work reported in this paper.

## Data Availability

Dataset for validating ENVI-met and assessing the benefits of facade greening during heatwaves (Original data) (Mendeley Data).
